# Small clonal B-cell population in the bone marrow as a possible tool in the diagnosis of occult primary parotid lymphoma

**Published:** 2016-03-30

**Authors:** Martha Romero, Guido R González-Fontal, Mónica Duarte, Carlos Saavedra, Andrés F Henao-Martínez

**Affiliations:** 1 Department of Pathology. Fundación Santa Fe de Bogotá, Bogotá, Colombia; 2 Division of Hemato-Oncology. Clínica Rey David, Cali, Colombia; 3 Hematology. Fundación Santa Fe de Bogotá, Bogotá, Colombia; 4 Division of Infectious Diseases, University of Colorado Denver, USA

**Keywords:** B-lymphocytes, bone marrow, diagnosis, lymphoma, parotid gland

## Abstract

**Case Description:**

An 82-years old Hispanic woman with a past medical history significant for pulmonary thromboembolism on oral anticoagulation, rheumatoid arthritis, and hypertension developed a new onset thrombocytopenia.

**Clinical Findings:**

Small clonal B-cells populations (SCBP) also known as monoclonal B-cell lymphocytosis was found as part of the workup for an idiopathic thrombocytopenia and lead ultimately to the diagnosis of parotid primary follicular lymphoma coexisting with Warthin tumor involving the bone marrow in a small extent and oncocytic papilloma located in the maxillary sinus.

**Treatment and Outcome:**

Patient was treated with Rituximab monotherapy with improvement on her platelet count.

**Clinical relevance:**

Although it is unclear the role of this clonal cells, they may work as a possible diagnostic tool for occult lymphomas. Further prospective studies are needed to confirm this possible association.

## Abstract (es)

**Descripción de caso:**

Mujer hispana de 82 años con una historia médica significativa de tromboembolismo pulmonar en anticoagulación, artritis reumatoide e hipertensión, la cual desarrolló recientemente una trombocitopenia

**Hallazgos clínicos:**

Una pequeña población de células B monoclonales también conocida como linfocitosis monoclonal de células B fue encontrado dentro del estudio de una trombocitopenia idiopática que conllevó al diagnóstico de un linfoma folicular primario de parótida coexistiendo con un tumor de Warthin y un papiloma oncocítico localizado en el seno maxilar.

**Tratamiento y resultado:**

La paciente fue tratada con monoterapia de Rituximab con una mejoría en su conteo de plaquetas.

**Relevancia clínica:**

Aunque el rol de las pequeñas poblaciones B monoclonales no está completamente dilucidado, podrían tener una aplicación como herramienta diagnóstica. Futuros estudios prospectivos son necesarios para confirmar esta posible asociación

## Introduction

The small clonal B-cells populations (SCBP) have been studied widely in peripheral blood. These have been detected with an estimated frequency between 3.5% and 14.0% in healthy subjects older than 40 years 1 and they are known as monoclonal B-cell lymphocytosis (MBL) 2. Its finding in peripheral blood is largely incidental, sometimes linked to some nonspecific clinical conditions 3,4 and in a minority of cases it confers an increased risk for the development of B-cell neoplasms 5,6. Nevertheless, the clinical significance of detecting an SCBP in bone marrow (BM) as a primary finding is largely uncertain at this time. Its evaluation in BM has been mainly used for lymphoma staging or to monitor response to treatment 7. But, the role as a useful diagnostic tool and in the clinical management of patients with occult lymphoma is not known. We present an unusual case of SCBP detection of 1% in BM by using a high sensitivity flow cytometry approach, during the evaluation of thrombocytopenia, which led to the diagnosis of a rare parotid follicular lymphoma associated with Warthin tumor which could not be found otherwise. The overall aim was to describe the possible association of SCBP with occult lymphomas.

## Case description 

An 82-years old Hispanic woman with a past medical history significant for pulmonary thromboembolism on oral anticoagulation, rheumatoid arthritis on treatment with hydroxychloroquine, and hypertension developed a new onset thrombocytopenia, with a platelet count of 52 x 10^9^/L during a routine assessment. She denied any symptoms and her physical exam was unremarkable. Complete blood count showed a hemoglobin of 14.6 g/dL; and a WBC count, 5.9 x 10^9^/L with 71% segmented neutrophils, 17% lymphocytes (absolute lymphocyte count: 1.0 x 10^9^/L), 11% monocytes, and 1% eosinophil. Her international normalized ratio was in a therapeutic range. Her initial thrombocytopenia evaluation based on the current guidelines 8 did not reveal the presence of an alternative diagnosis. Infectious disease testing including human immunodeficiency virus (HIV) was negative. Hence, given her age, a BM biopsy was performed. 

**Figure 1.**
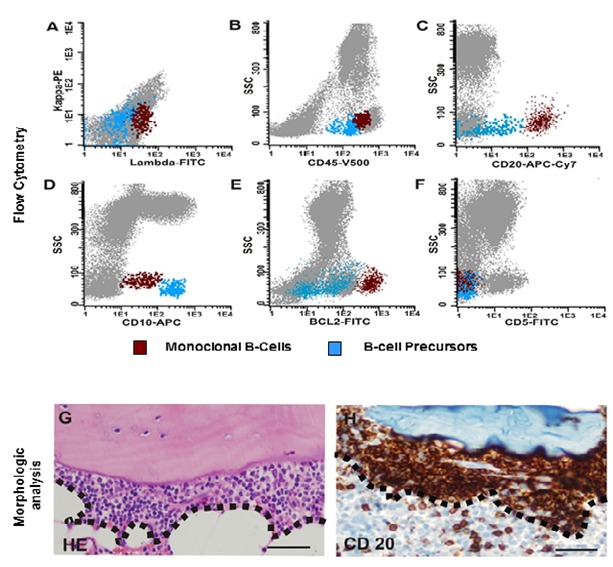
Bone marrow small clonal B-cells. Highly sensitive 8-color ﬂow cytometry approach identified 1% of lambda-restricted B cells **(A)** CD45+, CD20+, CD10+, BCL2++, CD5- **(B-F)** (SSC:side-scattered light; red population: monoclonal B-cells; blue population: precursor B-cell with normal antigen expression) Morphologic analysis revealed a 1% abnormal paratrabecular small cleaved lymphocyte infiltrate **(G)** (infiltration, surrounded by broken lines, HE: hematoxylin eosin staining). Immunohistochemistry staining demonstrated CD20 expression in paratrabecular tumor cells (original magniﬁcation for all photos x400 magnification, Bar= 50 µm).

Immunophenotypic studies of BM cells, using 8-color ﬂow cytometry approach (FACSCanto ll flow cytometer (BDB), and Infinicyt software program (Cytognos, V1.4)) detected 1% of Lambda-restricted B-cells CD45^+^, CD20^+^, CD10^+^, BCL2^++ ^(Fig. 1A-F), FMC7^+^, CD38^dim^, CD200^-^ and CD5^-^ (Fig. 1F). BM biopsy revealed a 1% paratrabecular small cleaved lymphocyte infiltrate. Immunohistochemical staining demonstrated CD20 (Fig. 1G-H), BCL2 and CD10 expression in tumor cells. 

**Figure 2.**
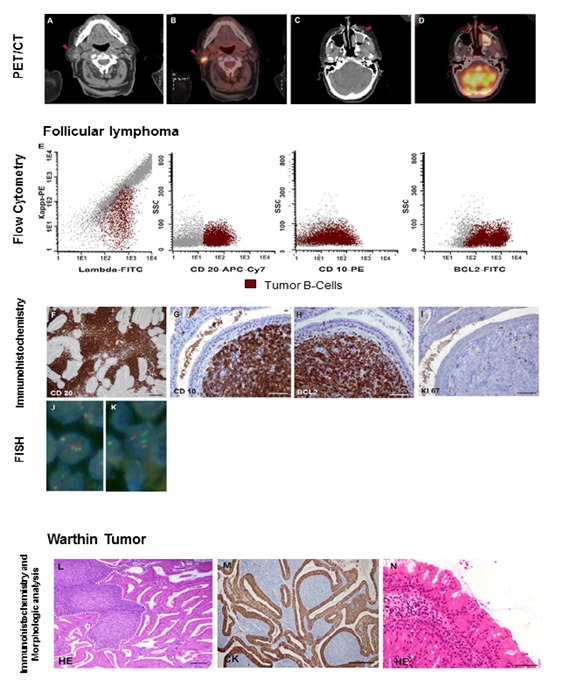
Coexistence of a parotid follicular lymphoma and Warthin tumor. PET/CT showed two mass-like foci (arrowheads on CT and fusion image) over right parotid **(A-B)** and left maxillary **(C-D)** with similar uptake. Parotid biopsy multiparameter flow cytometry showed lambda-restricted B-cells, CD20+, CD10+, BCL2+ **(E).** Histological analysis and immunochemistry confirmed a low-grade folicular lymphoma positive for CD20 (E, x200 magnification), CD10, BCL2, with low proliferation rate **(G-H**); **I**: Ki-67 immunostaining, (original magniﬁcation for all photos x400 magnification, Bar= 50 µm). Translocation (14; 18) was detected in BM biopsy and parotid lymphoma by FISH (**J**: Dual-fusion pattern in a balanced t (14; 18)(q32;q21) in parotid lymphoma cells). Normal signal pattern in epithelial cells is shown (**K**). Warthin Tumour composed of cystic spaces lined by oncocytic epithelium (**L-M**: follicular lymphoma: surrounded by broken white lines. CK: cytokeratin immunostaining) and oncocytic papilloma located over left maxillary **(N)** were also confirmed (original magniﬁcation for all photos x400 magnification, Bar= 50 µm).

Assessment with positron emission tomography/computed tomography (PET/CT) showed two mass-like foci over right parotid (Fig. 2A-B) and left maxillary (Fig. 2C-D) 

Parotid excisional biopsy multiparameter flow cytometry revealed a 42% of lambda-restricted B-cells, with low forward scatter (FSC) and SSC, CD20^+^, CD10^+^, BCL2^+^ (Fig. 2E), CD38^+^, CD19^+^, and CD45^+^; consistent with a diagnosis of follicular lymphoma. Examinations of the histological sections confirmed a low-grade follicular lymphoma, with a 5% proliferation rate measured by Ki-67 (Fig. 2F-I). Translocation t (14; 18) (q32; q21) was detected in parotid lymphoma (Fig. 2J) as well as BM biopsy, by using fluorescence in situ hybridization (IgH/ BCL2 dual color dual fusion translocation probes Vysis-Abbott).

The coexistence of a Warthin tumor composed of cystic spaces lined by papillary bilayered oncocytic epithelium was also observed (Fig. 2L-M). Pathologic examination of maxillary biopsy showed an oncocytic papilloma, constituted by a fibrovascular stroma lined by multiple layers of columnar cells with oncocytic features (Fig. 2N).

The final diagnosis was a parotid primary follicular lymphoma coexisting with Warthin tumor involving the BM in a small extent and oncocytic papilloma located in the maxillary sinus. Given her age, performance status and intermediate risk FLIPI (2 points) she was treated with Rituximab monotherapy, having received four cycles at the last clinical follow-up with improvement on her platelet count up to 165 x 10^9 ^/L.

### Consent

Written informed consent was obtained from the patient for publication of this case report and accompanying images.

## Discussion 

The SCBP in peripheral blood behaves as a transient population, which might be associated with the normal process of immunosenescence in older people. It is also postulated that can be triggered by infectious agents that can cause chronic and persistent antigenic stimulation 9. The majority of MBL (75%) shows similar phenotypic characteristics to chronic lymphocytic leukemia (CLL) cells and the remaining are classified as atypical CLL and CD5-negative MBL 10, and least frequent, CD10+ B-cell clones 1.

SCBP have been detected in myelodysplastic syndrome, post chemotherapy and benign disorders such as immune related or isolated thrombocytopenia, chronic obstructive pulmonary disease, and chronic cardiovascular disease 11 and in 1% of cases will be precursor of B-cell neoplasm 5,6. It is unclear if rheumatoid arthritis or its treatment played a role in the onset of the clonal cells or the tumor.

In the only available retrospective cohort review of patients with incidental documentation of SCBP detected in BM; 29% of patients developed non-Hodgkin lymphoma (NHL) 11 in up to 40 months of follow up. Three patients were classified under the CD5-/CD10+ immunophenotype; from them, one developed NHL. Likewise, in the same study there were some limited associations with diffuse large B-cell lymphoma, hairy cell leukemia, splenic B-cell marginal zone lymphoma, and Waldenström macroglobulinemia. In our case, it led to the diagnosis of a rare parotid follicular lymphoma associated with Warthin tumor, an unusual coexistence, with 23 previous cases reported.

Herein we were able to establish an association of SCBP in BM with a glandular based lymphoma. The finding of this type of population of cells prompted further workup that ultimately led to the final diagnosis. It is unclear the clinical significance of these clones and it still can be a serendipitous association; however they proved to be successful detecting varies forms of NHL, as pointed out in our case. Interestingly, in the previous report by Chen *et al*., what initially prompted further analysis in the majority of cases was cytopenias 11. Similarly, the thrombocytopenia was the initially detected abnormality in this case. There is insufficient evidence to use these clone cells in BM as a potential screening tool for lymphomas or to consider them a real premalignant condition. Further prospective studies may prove useful to confirm the clinical utility of this particular finding.
